# Telehealth in Colombia, challenges associated with COVID-19

**DOI:** 10.7705/biomedica.5594

**Published:** 2020-11-12

**Authors:** Luis Mauricio Figueroa

**Affiliations:** 1 Sección de Cirugía Pediátrica, Departamento de Cirugía, Escuela de Medicina, Universidad del Valle, Cali, Colombia Universidad del Valle Departamento de Cirugía Universidad del Valle Cali Colombia

**Keywords:** Coronavirus infections, epidemics, telemedicine, health education, information technology, infecciones por coronavirus, epidemias, telemedicina, educación en salud, tecnología de la información

## Abstract

The COVID-19 pandemic has generated a revolution of such magnitude that no aspect of human life will be the same from now on. The provision of health services and health education are not unrelated to this new normality imposed by the disease, and its consequences have been reflected in the need to use protocols and resources based on virtuality that most of us had not valued in their real dimension. Telehealth and telemedicine will be basic tools for professionals and teachers and it is our obligation to know them, apply them, and innovate to adapt to this reality.

## Resumo

La pandemia de COVID-19 ha generado una revolución de tal magnitud que ningún aspecto de la vida del ser humano será igual a partir de ahora. La prestación de los servicios de salud y la educación en salud no son ajenas a esta nueva normalidad impuesta por la enfermedad, y sus consecuencias se han visto reflejadas en la necesidad de utilizar protocolos y recursos basados en la virtualidad que la mayoría no habíamos valorado en su real dimensión. La telesalud y la telemedicina serán herramientas básicas para profesionales y docentes y es nuestra obligación conocerlas, aplicarlas e innovar para adaptarnos a esta realidad.

The transmission of medical information through different means of communication has been a need since the antiquity. At the time of the bubonic plague in Europe, the heliograph was used; during the American Civil War, the telegraph, and during the First World War, the field telephone.

The year 2002 marked the 100^th^ anniversary of the refinement of the electrocardiography machine and its first transmissions by the physician, physiologist, and winner of the Nobel Prize in Medicine Willem Einthoven. He was the first clinical scientist to develop, in the most modern sense of the word, a systematic telehealth technique ^1^.

In the United States, the pioneers in distance learning were the physicians Cecil Wittson and Reba Benschoter from the University of Nebraska Medical Center. Doctor Wittson conceived the first two-way closed-circuit television system in the United States developed and tested through grant support secured in 1963 ^2^. This television system made possible face-to-face communication between the Nebraska Psychiatric Institute (NPI), located on the medical campus in Omaha (now the site of Durham Research Center towers) and the Norfolk State Mental Hospital, located in Norfolk 112 miles away ^3^.

The term telehealth (assisted healing) was coined by Thomas Bird in 1970 and its progress was marked by two major milestones: The development of the telecommunications and the space race ^4^. Although there are numerous publications in the world about the use of telehealth in different specializations and online education in different programs in the field of health, in Colombia the first projects were only structured in the XXI century by institutions such as *Universidad de Caldas, Universidad Nacional de Colombia, Universidad del Cauca,* and *Universidad de Antioquia,* among others, and by foundations such as *Fundación Santa Fe de Bogotá* and *Fundación Cardiovascular de Bucaramanga*^5^.

Despite the demonstrated benefits of these care strategies for patient access to health services, reduction of costs, and the rapprochement by institutions and specialists to distant communities, obstacles such as the absence of technological resources and communications in the regions and the lack of training in the operation of digital platforms, among others, have deterred the dissemination of telehealth throughout the country. Regarding online education, universities have tried to translate many of their contents and offer programs through online education but not many of the educators and students are familiarized with these resources or they do not have access to technological devices and means of communication such as the internet ^6^.

Until 2010, there were only 43 research or health service-provision telehealth projects in Colombia and they benefitted only 550,000 people and 650 healthcare institutions, which is a rather poor offer in a country as large and diverse where most of its almost 50 million inhabitants have low income and would be greatly benefited by these strategies ^7^. Although today the offer of telehealth and online education programs has increased in Colombia and there is strong legislative support ^8^, the flaws of such programs have become evident after the COVID-19 pandemic.

Healthcare centers, higher education institutions tasked with the training of healthcare personnel, and government entities have to endeavor in an urgent and joint effort to invest in the qualification of human resources, the infrastructure, the deployment of connectivity networks, the endowment of technological resources (software and hardware), and the acquisition of communication devices ^9^ to ensure the population's access to healthcare services via teleconsultation and, thus, avoid breaking isolation and social distancing that may expose patients and caregivers to infection.

Regarding health education, resources like simulation, online education, telementoring, telepresence, and telesupervision should help students to obtain the necessary knowledge and achieve the competencies they will need as professionals despite the difficulties springing from the need to temporarily marginalize from the experience of direct training practices due to the pandemic by reducing contact with patients in real life during their instruction ^10^^,^^11^.

This is possible, as shown by the progress achieved by institutions such as the *Universidad Nacional de Colombia* and *Hospital Universitario Nacional de Colombia* in Bogotá, which have already taken the first steps and are formulating strategies like the "TeleUCI" (remote intensive care unit) with the participation of an interdisciplinary team made up of specialists in critical care, physiotherapists, and nurses intent on creating the first national telemedicine unit in intensive care for adults to assist referral centers for distant populations that do not have enough human resources to face the increase in patients due to the pandemic.

It is the responsibility of health professionals to adapt and plan innovative strategies and change existing paradigms ^9^^,^^12^ vis-á-vis the new challenges patients may pose as a result of their updated access to information. The same should apply to the field of education as institutions have to ensure the quality of curricula, biosafety, competencies by area, and lower risks for students and professors.

Finally, it is a must that the support from government entities be reflected in the necessary modifications allowing universities and health service providers to streamline their link to this different, but not new, form of education and care without affecting patients' privacy, information management, and the quality of services ^13^.
